# Paedomorphosis and retention of juvenile diet lead speciation in a group of Neotropical snakes (Colubroides-Philodryadini)

**DOI:** 10.1038/s41598-024-60885-y

**Published:** 2024-05-02

**Authors:** Mariana Chuliver, Agustín Scanferla

**Affiliations:** 1 Fundación de Historia Natural “Félix de Azara”, Hidalgo 775, C1405BCK Ciudad Autónoma de Buenos Aires, Argentina; 2 Consejo Nacional de Investigaciones Científicas y Técnicas (CONICET), Ciudad Autónoma de Buenos Aires, Argentina

**Keywords:** Evolution, Herpetology

## Abstract

Dipsadidae is one of the largest clades of extant reptiles, showing an impressive morphological and ecological diversity. Despite this fact, the developmental processes behind its diversity are still largely unknown. In this study, we used 3D reconstructions based on micro-CT data and geometric morphometrics to evaluate the skull morphology of *Philodryas agassizii*, a small, surface-dwelling dipsadid that consume spiders. Adult individuals of *P. agassizii* exhibit a cranial morphology frequently observed in juveniles of other surface-dwelling colubroideans, represented in our analysis by its close relative *Philodryas patagoniensis*. Large orbits, gibbous neurocranial roof and a relatively short jaw complex are features present in juveniles of the latter species. Furthermore, we performed an extensive survey about diet of *P. patagoniensis* in which we detected an ontogenetic dietary shift, indicating that arthropods are more frequently consumed by juveniles of this dietary generalist. Thus, we infer that *P. agassizzii* retained not only the ancestral juvenile skull morphology but also dietary preferences. This study reveals that morphological changes driven by heterochronic changes, specifically paedomorphosis, influenced the retention of ancestral life history traits in *P. agassizii*, and therefore promoted cladogenesis. In this way, we obtained first evidence that heterochronic processes lead speciation in the snake megadiverse clade Dipsadidae.

## Introduction

Dipsadidae stands out as one of the largest family of extant reptiles (> 800 species) with nearly all its representatives restricted to the New World^1,2^. Their species richness is paralleled by their ecological diversity, ranging from aquatic, arboreal, terrestrial and cryptozoic, and including an enormous variety of diets^1,3^. Despite the relevance of this clade for the understanding of the adaptive radiations and cladogenetic events that shaped colubroideans (Colubroides sensu^4^), the developmental processes underlying the morphological diversity within the group are still largely unknown.

**Figure 1:**
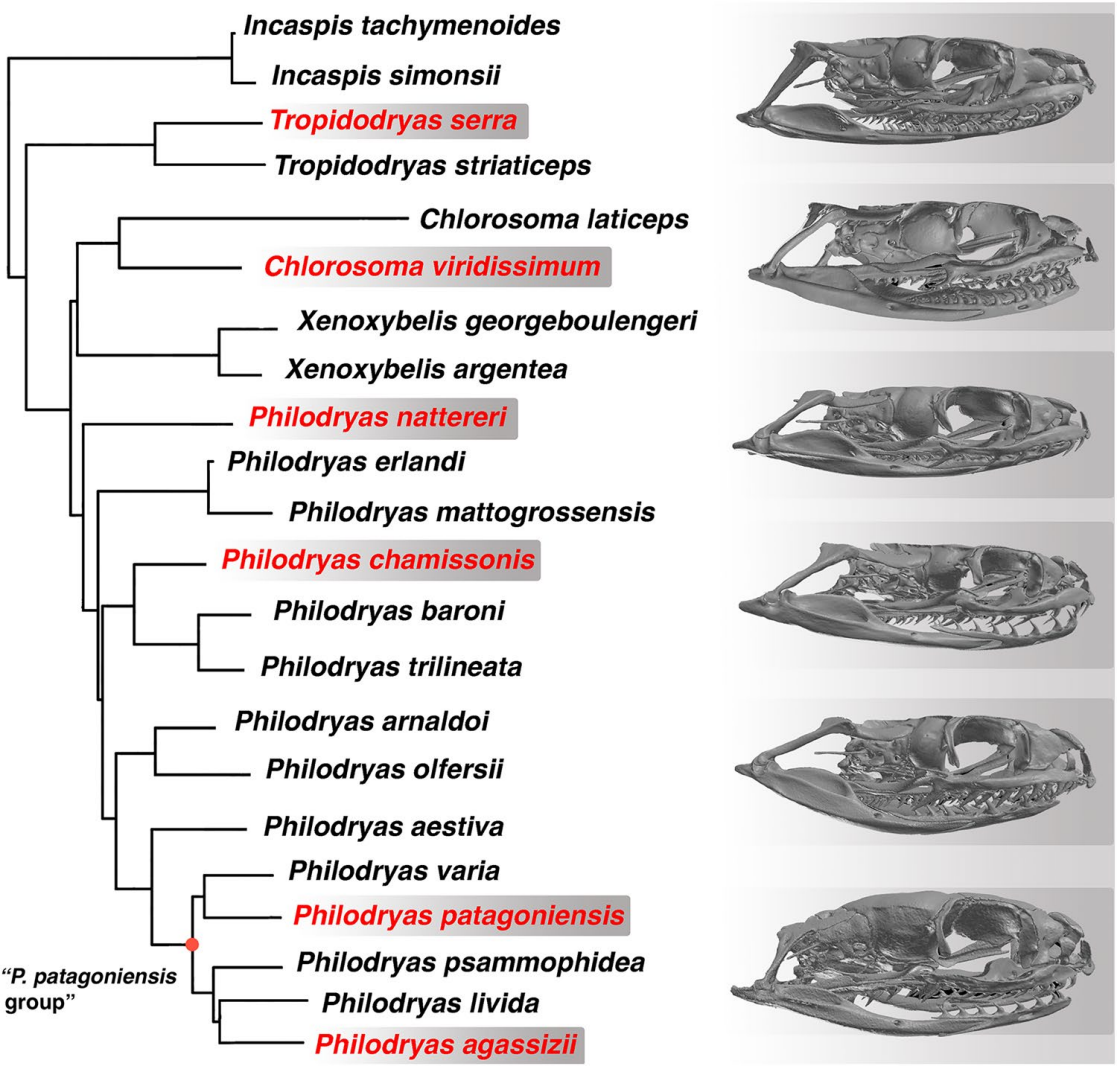
Phylogenetic tree of Philodryadini (modified from^5^). The adult skull morphology of selected species (names in red) is shown on the right panel. Note the contrasting shape of the adult skull of *Philodryas agassizii* in comparison with the rest of species. Skulls are not in scale.

South American racers of the tribe Philodryadini constitute a diverse clade among dipsadids^5,6^. The 26 species recognized in this clade display an array of ecomorphologies ranging from terrestrial to fully arboreal forms including both harmless and venomous species^7,8^. Nearly all species of this clade are large snakes that surpass 1 m body length and consume a vast array of different vertebrate prey^8,9^. Notably, *Philodryas agassizii* represents an exception to this generalization, as this terrestrial species is the smallest among philodryadines (400–500 mm TL) and prey almost exclusively upon arthropods, mostly spiders^7^. This species also departs from the overall morphology of philodryadines due to its short tail and an antero-posterior reduction of dorsal scale rows^7^. Moreover, a recent work points out the particular nature of the venom of *Philodryas agassizii*, thus highlighting the distinctiveness of this species among the clade Philodryadini^10^. Philodryadines have a long-debated taxonomy^1,11–13^, although recent efforts have been made in order to unravel their interrelationships^5,6^. The aforementioned works emphasizes the close relationships between *P. agassizii* and the Patagonian green racer *Philodryas patagoniensis*, forming a small clade of basal philodryadines^6^ or included in a larger subclade informally dubbed as “*P. patagoniensis* group”^5^ (Fig. 1).

Studies of postnatal ontogeny can be crucial in revealing new information about the evolution of morphological novelties in snakes^14–17^. Moreover, there is an increasing body of information on the interplay between postnatal skull transformations and ontogenetic dietary shifts^18–20^. As shifts in dietary ecology are important drivers of adaptive radiation, knowledge about snake diet sheds light into evolutionary mechanisms of adaptively radiating clades^21–23^.

During a survey of cranial ontogeny of snakes we found that *P. agassizii* departs from the typical morphology seen among its congeners, displaying features observed in juvenile individuals of many colubroidean species, such as a relatively short gnathic complex (i.e., palatomaxillary bar, suspensorium and lower jaw) and a gibbous neurocranium (Fig. 1). In order to interpret this observation, we explore the cranial anatomy of the species of Philodryadini, emphasizing the postnatal transformations of the skull of *P. agassizii* in comparison with species of the “*P. patagoniensis*” group*.* For this purpose, we addressed the postnatal ontogeny of the closely related species *P. patagoniensis*. Using micro-CT scanning to study skull ontogeny we track the pathway of several cranial features to determine the polarity of morphological change, and identify the developmental processes most likely to be operating. Also, we perform a dietary analysis of *P. patagoniensis* in order to find feeding patterns and/or variations along ontogeny related to size and skull morphology. Through this, we aim to track evolutionary changes in developmental processes giving rise to *P. agassizii* morphology and feeding habits.

## Results

### Skull anatomy of *Philodryas agassizii*

**Figure 2:**
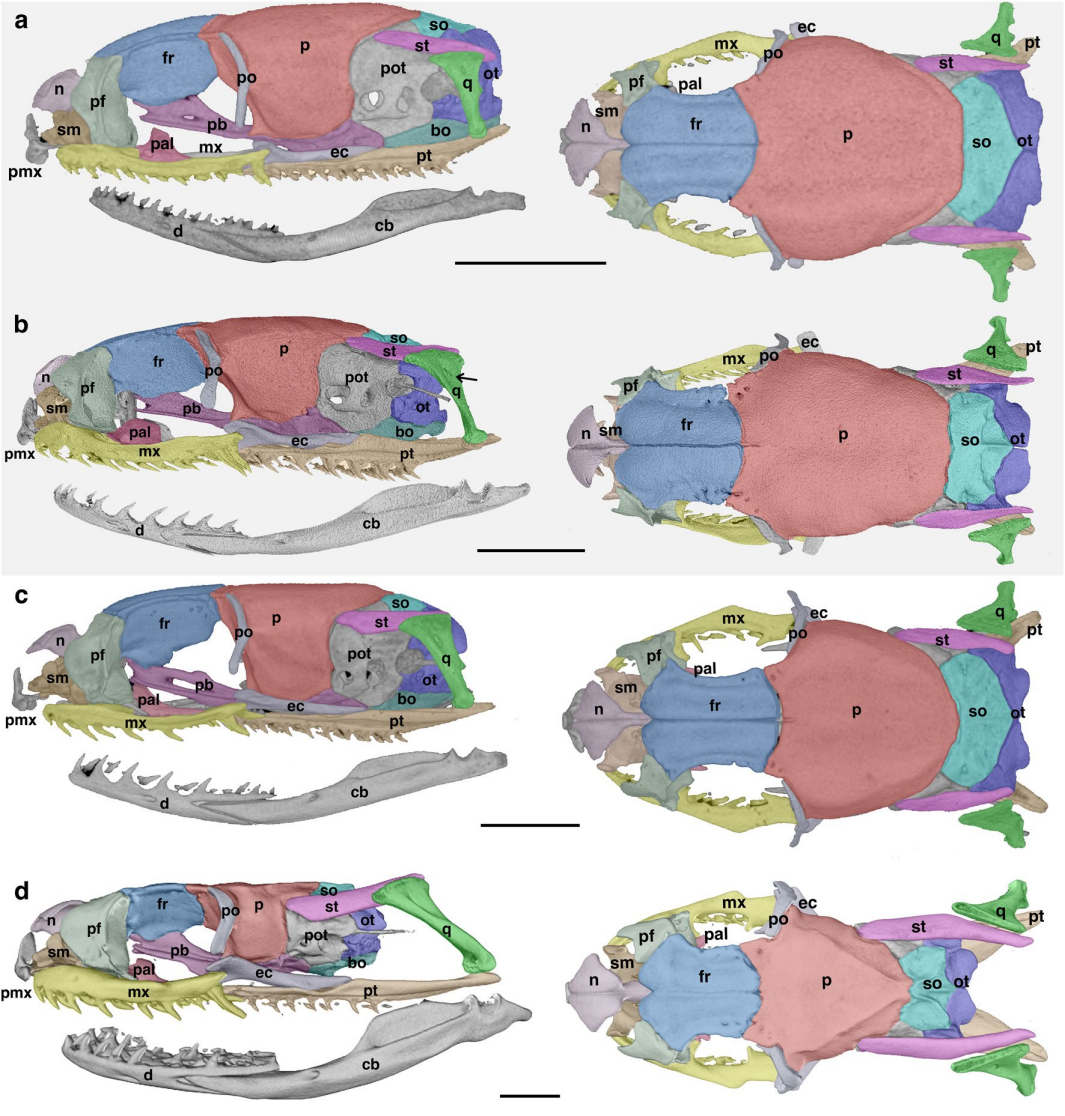
Comparisons of skull of juvenile (**a**, TL = 196 mm) and adult (**b**, TL 382 mm) of *Philodryas agassizii*, and juvenile (**c**, TL = 266 mm) and adult (**d**, TL = 952 mm) of *Philodryas patagoniensis*. *bo* basioccipital, *cb* compound bone, *d* dentary, *ec* ectopterygoid, *fr* frontal, *mx* maxilla, *n* nasal, *ot* otooccipital, *p* parietal, *pal* palatine, *pb* parabasisphenoid, *pf* prefrontal, *pmx* premaxilla, *po* postorbital, *pot* prootic, *pt* pterygoid, *q* quadrate, *sm* septomaxilla, *so* supraoccipital, *st* supratemporal. The arrow points out the quadrate foramen. Scale bars equal to 2.5 mm.

**Figure 3:**
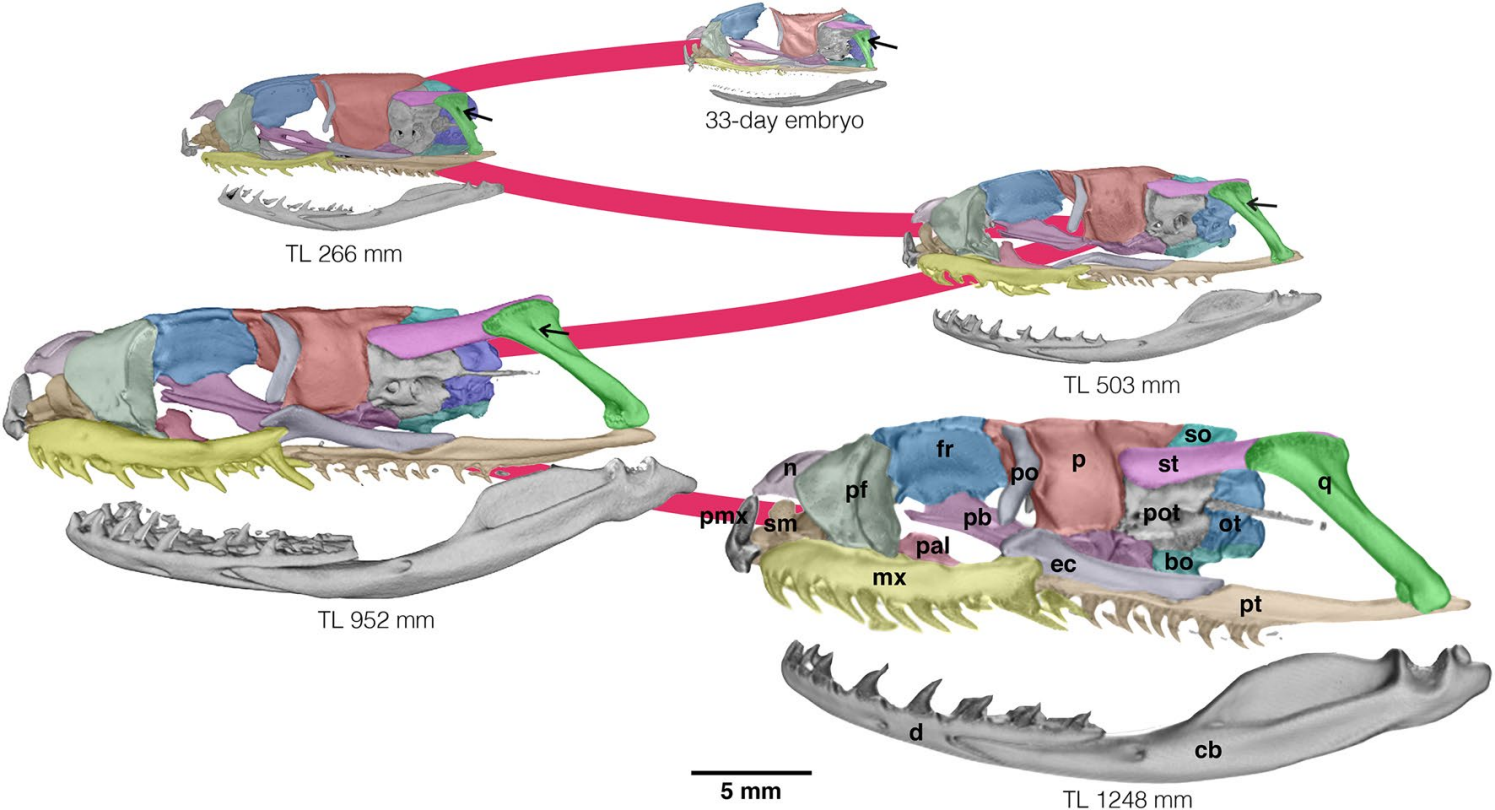
Postnatal series of *Philodryas patagoniensis* spanning from a late embryo to the largest specimen analyzed. *bo* basioccipital, *cb* compound bone, *d* dentary, *ec* ectopterygoid, *fr* frontal, *mx* maxilla, *n* nasal, *ot* otooccipital, *p* parietal, *pal* palatine, *pb* parabasisphenoid, *pf* prefrontal, *pmx* premaxilla, *po* postorbital, *pot* prootic, *pt* pterygoid, *q* quadrate, *sm* septomaxilla, *so* supraoccipital, *st* supratemporal. The arrow points out the quadrate foramen.

**Figure 4:**
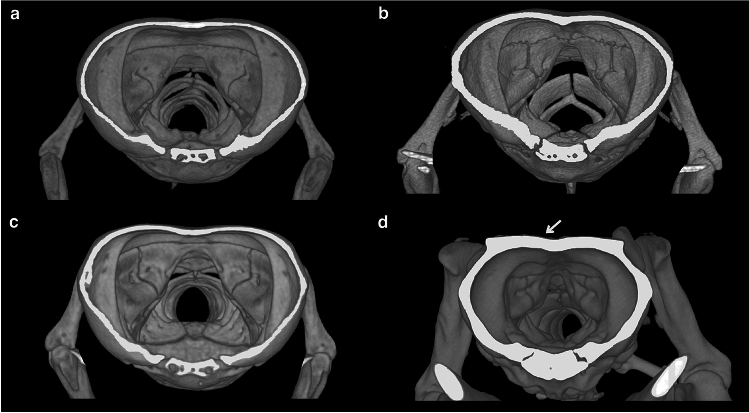
Transversal cutaway posterior to the postorbital bone depicting the thickness of the parietal bone in 3D reconstructions of the skull of juvenile (**a**, TL = 196 mm) and adult (**b**, TL 382 mm) *Philodryas agassizii*, and juvenile (**c**, TL = 266 mm) and adult (**d**, TL = 952 mm) *Philodryas patagoniensis*. The arrow points out the parietal table. Images not to scale.

The adult skull morphology of *Philodryas agassizii* (TL 382 mm) departs from the adult skull pattern shared by most species of philodryadines (Fig. 1) and many other surface-dwelling colubroideans^14–16^. Comparisons between *P. agassizii* and its close related congener *Philodryas patagoniensis* indicate they follow different ontogenetic postnatal trajectories (Fig. 2). In *P. agassizii* adult the orbits are relatively large with respect to the rest of the skull (Fig. 2). This clearly affects the shape of the dorsal surface of the maxilla, which retains the marked concave shape present in newborn and juvenile specimens (TL < 400 mm) of *P. patagoniensis* (Figs. 2, 3). Both, juvenile and adult *P. agassizii* share with juvenile *P. patagoniensis* a general gibbous aspect of the neurocranium due to the absence of the parietal table and the poor development of crests in the neurocranial roof. During postnatal ontogeny, the braincase roof of *P. patagoniensis* experience an increase in thickness, alongside with a marked development of bony crests (Figs. 3, 4). The parietal table is then configurated in adults of *P. patagoniensis*, with the ossification of crests for attachment of adductor musculature as in most colubroideans (Fig. 4).

The most remarkable traits in the skull of *P. agassizii* are related to the gnathic complex (i.e., palatomaxillary bar, suspensorium and lower jaw). Despite in *P. agassizii* there is positive allometric growth of the gnathic complex with respect to the rest of the skull during early stages of postnatal development, its developmental trajectory is arrested at an ontogenetic stage morphologically equivalent to juvenile stages of other philodryadines. Therefore, the adult gnathic complex of *P. agassizii* resembles the condition present in juveniles (TL 400–500 mm) of *P. patagoniensis* (Figs. 2, 3). Accordingly, the supratemporal of *P. agassizii* barely surpasses the posterior limit of the braincase marked by the posterior border of the otooccipital, and therefore its free-ending process is weakly developed. The quadrate bone has a short shaft when compared to adult *P. patagoniensis*, and retains a large quadrate foramen in its lateral face (Fig. 2), typical of early stages of development of most alethinophidian snakes (pers. obs.). The same tendency is observed in the palatomaxillary bar and lower jaw due to the weak growth of the quadrate ramus of the pterygoid and the compound bone. Thus, the mandibular articulation slightly surpasses the posterior limit of the skull marked by the occipital condyle (Fig. 2). On the contrary, in *P. patagoniensis* the elements of the gnathic complex experience positive allometric growth throughout postnatal ontogeny, and they tend to be extremely elongated with respect to the rest of the skull in specimens with large body length (TL > 900 mm). The quadrate bone also shows a significant growth through the elongation of the quadrate shaft and the anteroposterior expansion of its cephalic head. The quadrate foramen progressively diminishes its size and it is completely close in adult specimens (Fig. 3).

### 3D geometric morphometric analysis

**Figure 5:**
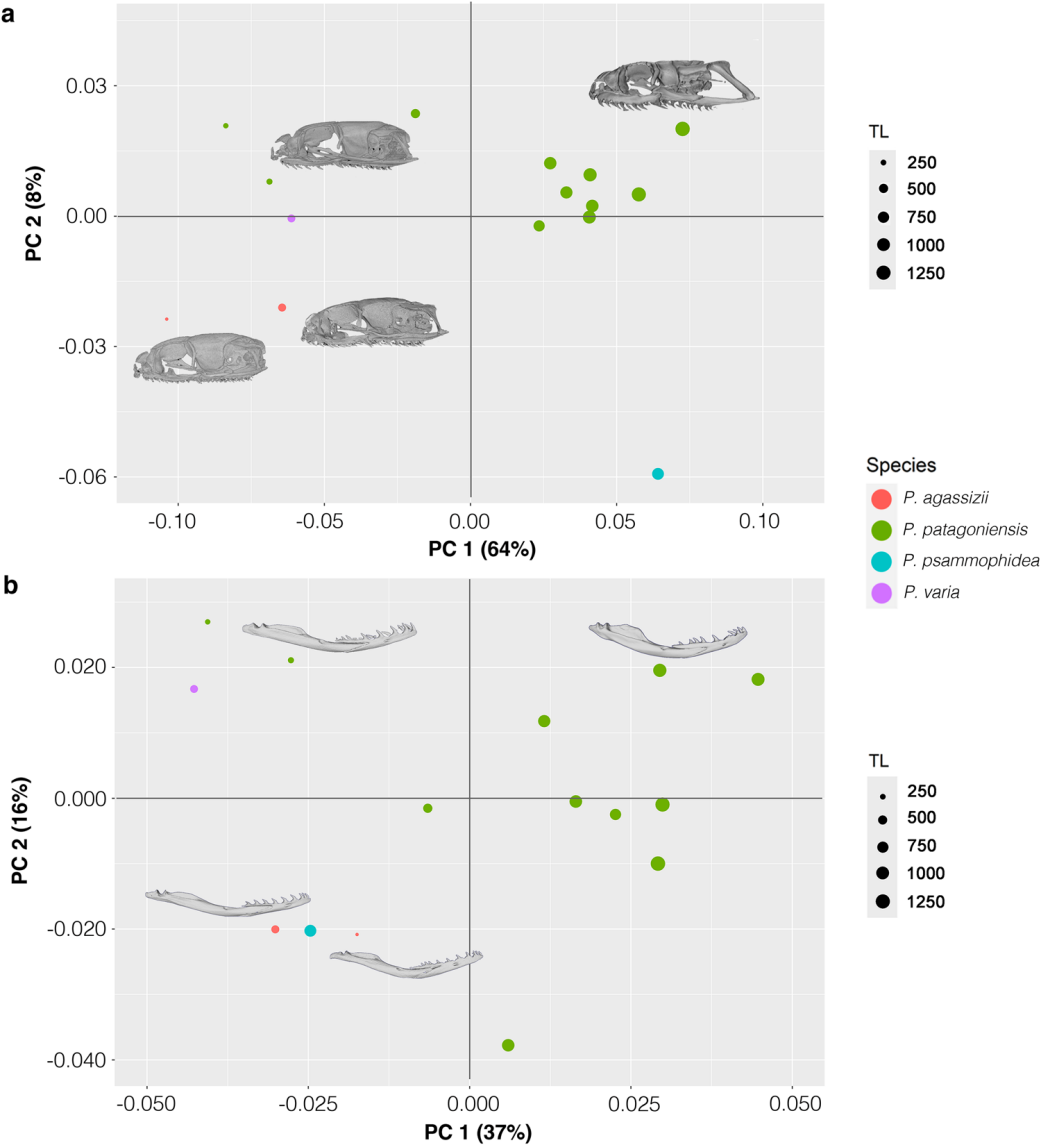
Cranial and mandibular morphospaces of four species of *Philodryas*. (**a**) PCA plot of the first two principal components (PCs) of the skull. (**b**) PCA plot of the first two principal components of the lower jaw. The actual shapes of *P. patagoniensis* juvenile and adult, and *P. agassizii* juvenile and adult are drawn next to the corresponding points.

The performed 3D geometric morphometric analysis of the skull shows that the Procrustes distance between adult *P. agassizii* and juvenile *P. patagoniesis* (TL 266 mm) is lesser than the distance between the latter and the smallest adult of *P. patagoniesis* (TL 737 mm; Supplementary Table S5). In this sense, our PCA analysis shows that the overall skull shape of the adult of *P. agassizii* strongly matches with that of early postnatal stages of *P. patagoniensis* and of *Philodryas varia* (TL 370 mm; Fig. 5). The PCA of the cranium indicates that the first principal component (PC1) accounts for 64% of the total variance, and according to the *getMeaningfulPCs* function it was the only axis meaningful to describe the total shape variation (Fig. 5A). The PC1 separates the individuals of *P. patagoniensis* ontogenetically, being adults located at positive values of PC1, while the early postnatal stages (i.e. newborns and juveniles) along with specimens of *P. agassizii* and juvenile of *P. varia* are located at negative PC1 values (Fig. 5A). Positive skull shape variations along the PC1 axis involve elongation of elements of the gnathic complex (supratemporal and pterygoid), and narrowing of the posterior region of the skull roof due to remodeling and growing of crests of parietal and supraoccipital (Fig. 5A).

Similarly, the lower jaw shape analysis indicates low Procrustes distance between adult of *P. agassizii* and juvenile of *P. patagoniesis* (TL 503 mm) and *P. varia* (TL 370 mm), while this distance is bigger between the smalles juvenile specimen of *P. patagoniensis* (TL 266 mm) and adults of the species (Supplementary Table S6)*.* The PCA of the lower jaw shows the most important shape variations along PC1 (37%), being the specimens of *P. patagoniensis* distributed ontogenetically along this axis (Fig. 5B). Interestingly, both juvenile and adult of *P. agassizii* are clustered with *Philodryas psammophidea* adult on the negative values of both PC axis. The PC1 values concentrate changes in curvature of the compound bone, height of the prearticular crest, and angle of articulation between dentary and compound bone (Fig. 5B). Changes along PC2 represent 16% of the total shape variation and involve mainly deflection of the anterior tip of the dentary (Fig. 5B).

### Ontogenetic dietary survey of *Philodryas patagoniensis*

**Figure 6:**
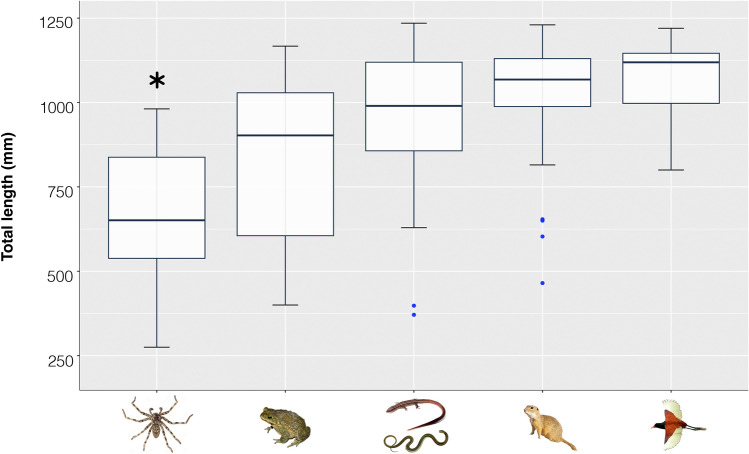
Box-plot graph of the ANOVA comparing prey items against total length of the 145 surveyed specimens of *Philodryas patagoniensis*. The variable marked with an asterisk is significantly different (Tukey post hoc tests, p < 0.05) from all other prey categories.

Our dietary survey includes 145 records covering most of the species geographic distribution (Supplementary Table S7), thus being more comprehensive than previous published data^9,24,25^. We recognized 20 prey items from two clades of arthropods (Arachnida and Insecta; prey type I of^26^) and 125 prey items from vertebrate clades (Anura, Squamata, Aves, and Mammalia; Fig. 6). Squamate reptiles, including lizards and elongated forms such as snakes and limbless lizards, were the most consumed prey (49 items; 33.8%), followed by anuran amphibians (36 items; 24.8%), and mammals (27 items; 18.6%), being birds the least consumed item (12 items; 8.3%). Anurans are consumed by the largest range of body lengths (TL ranging from 400 to 1354 mm). We found significant differences among total length of individuals with respect to prey type (F = 10.63, p < 0.0001), and post hoc test revealed significant differences between TL of those specimens consuming arthropods and all other prey categories (Supplementary Table S8). Finally, mammals (with few outliers) and birds were mainly consumed by large adult individuals, spiders and insects were mostly consumed by small individuals (TL < 700 mm; Fig. 6).

## Discussion

### Skull anatomy

During postnatal development most snake species experience remarkable morphological changes, since newborns not only differ in absolute size from adults, but also skull shape is markedly modified and cranial elements may follow independent growth trajectories^15–18,27^. However, the anatomical analyses presented here indicate that the juvenile and adult of *Philodryas agassizii* show similar skull morphologies, evidenced by its proximity in morphospace. Moreover, the overall skull morphology of adult *P. agassizii* strongly resembles that of juveniles *Philodryas patagoniensis* and *Philodryas varia* (Fig. S2), as well as other surface-dwelling colubroidean snakes^14–16,20^.

Particularly, the adult morphology of the gnathic complex of *P. agassizii* appears to be consequence of the early truncation of the postnatal allometric growth of its elements in contrast to the ontogeny of the putative ancestor, as observed in other philodryadines. Bony elements of the gnathic complex (i.e. palatomaxillary bars, suspensorium and lower jaw) exhibit positive allometric growth during postnatal ontogeny in many surface-dwelling colubroidean snakes^14–16,18,20^. These changes are correlated to a dietary shift from juveniles to adult forms, allowing the macrostomous condition (i.e. ingestion of large prey with a large cross-sectional area in relation to the head dimensions of the snake^28,29^). Accordingly, many of the characters of the gnathic complex that represent the distinctive traits of the macrostomous snake skull are weakly developed in *P. agassizii.* This is probably tightly related to the specialized diet of the species, since reduction or loss of macrostomy is also correlated to the absence of ontogenetic dietary shift^15,29^. These modifications in the jaw complex elements correlated to a small gape, are recurrent in extant clades of colubroidean snakes exploiting underground habitats^15^. Notably, data presented here represent the first record of a surface-dwelling species with this particular cranial anatomy, suggesting that other driving forces different than those exerted by underground life were involved in the loss of bony conditions for macrostomy among colubroidean snakes.

In this sense, Klaczko et al.^30^ in their pioneer work employing 2D geometric morphometrics found that skull morphology of a small sample of dipsadid snakes was highly associated with their diet. Recently, Pandelis et al.^3^ found that ecological factors, including diet, contribute to overall variation in skull shape across dipsadines. Notably, they acknowledged that developmental constraints may play a more dominant role in explaining skull morphology than the variables tested in their work. Herein, we provide evidence for this assertion, thus suggesting that heterochrony played a major role in the evolution of skull morphology in dipsadid snakes.

### Diet

Our dietary survey indicates that *Philodryas patagoniensis* is a generalist that experiments an ontogenetic dietary shift as consumption of relatively smaller items (such as spiders and insects) decrease significantly as they grow, in accordance with previous studies for the species^9,25,31^. Remarkably, most *Philodryas* species exhibit generalized feeding habits including a wide variety of vertebrate preys, thus suggesting that a generalist diet represents the ancestral state for the clade Philodryadini^8^. Hence, *P. agassizii* outstands as a dietary specialist among philodryadines^7,8^, and we infer that it represents a retention of the ancestral juvenile diet.

Dietary ontogenetic shifts associated with skull transformations are common for alethinophidian snakes^15,18–20,32,33^, but also juvenile and adult venom composition could differ substantially in its biological activity on different prey types^34,35^. Notably, a recent venomic analysis among philodryadines revealed a particular venom composition for *P. agassizii* with respect to the rest of species of *Philodryas*^10^. Its venom exhibits a particular proportion of proteic components*,* including a high proportion of non-enzymatic proteins 3FTXs superfamily, which can be taxon-specific and thus targeting specific types of prey^36^. Despite there is no information about ontogenetic changes in venom composition of philodryadines, the retention of juvenile traits in skull anatomy and diet specialization detailed above suggest that differences in venom composition of *P. agassizii* could also be explained by retention of the juvenile venom composition in adults. Retention of ancestral juvenile venom composition has been reported previously in other colubroidean snakes of the family Viperidae, indicating that heterochronic processes have played a relevant role in venom composition^37–39^. Among viperid species, the insular lancehead *Bothrops alcatraz* represents an analogous to *P. agassizii*, since this species experiences morphological changes associated with heterochronic processes when compared to its ancestral continental species *Bothrops jararaca*. Moreover, *B. alcatraz* feeds on arthropods and lizards, thus also retaining the juvenile diet present in *B. jararaca*^40^. Further venomic analyses of philodryadines focusing on potential ontogenetic changes are needed in order to test this hypothesis.

### Paedomorphosis in the evolution of colubroidean snakes

Paedomorphosis is a heterochronic process defined as the displacement of ancestral juvenile features to adult stages of the descendants^41–43^. We have identified several distinctive features of *Philodryas agassizii* that depart from other representatives of the clade Philodryadini and other surface-dwelling colubroideans. Our qualitative and quantitative morphological analyses of the postnatal ontogeny indicate that these features resemble early postnatal stages of the ancestral ontogeny, exemplified here by *Philodryas patagoniensis.* Hence, the adult skull morphology and dietary pattern observed in *P. agassizii* appears to be generated by premature cessation of growth in this species relative to the ontogeny of the putative ancestor, and thus indicating that paedomorphosis had a major role on its evolution.

Taking into account that paedomorphosis entails evolutionary consequences on trophic ecology^41,44,45^ we inferred that the combination of paedomorphic skull anatomy, diet and probably venom composition are likely to have played key roles in the speciation of *P. agassizii* by causing a niche differentiation. That is, these attributes could promote new ecological opportunities through a lower resource competition with the ancestral phenotype*.* It is important to note that recent works indicated that species of different colubroidean snake clades that acquired a diet specialization are smaller than the ancestral related diet generalists^46,47^. Therefore, we suggest that trophic niche differentiation via body size reduction associated with morphological novelties in the skull triggered by paedomorphosis may be relevant for dipsadid snakes evolution. Probably, diet specialization mediated by the aforementioned anatomical changes have had a relevant role as a covariant trait in the evolution of these clades, not only in cryptozoic forms but also small surface-dwelling species.

We emphasize that this hypothesis should be considered and tested using integrative analyses of morphological and ecological traits that include numerous specimens*.* Assembling ontogenetic series of snake species, and the broad access to osteological data through massive repositories of micro-CT data will allow to identify potentially paedomorphic forms among dipsadids and other colubroidean clades, and therefore, will provide further insights into the role of heterochrony in colubroidean snakes evolution.

## Methods

### Specimens

Our survey on cranial morphology comprised fourteen species of Philodryadini, including dry skeletons and x-ray micro-computed tomography (micro-CT) data (Supplementary Tables S1). Furthermore, fifteen specimens representing different ontogenetic stages of four species of *Philodryas* (*P. agassizii*, *P. patagoniensis*, *P. varia* and *P. psammophidea*) were comprehensively analysed through a 3D geometric morphometric approach (Supplementary Table S2). The snout-vent length (SVL) and total length (TL) of each specimen was registered. Specimens were divided into three age classes that broadly reflect ontogenetic differences: neonate, juvenile, and adult (Supplementary Table S2). Individuals were categorized as: adults if they had a TL equal to or over 700 mm, as this is the average size reported for sexual maturity in different populations of *P. patagoniensis*^9,24,25,48^; juveniles if their TL was lower than 700 mm; neonates if they retain egg tooth and exhibited umbilical scar that persists for few days to weeks in colubroidean snakes^49,50^. Due to the significant differences between sexes in jaw complex structures (e.g.^51^), we chose females for the study. Descriptions of the skulls and their elements follows the terminology of Cundall and Irish^28^.

### Micro-CT

3D reconstructions of skulls from micro-CT data were downloaded from the Morphosource database (https://www.morphosource.org/) or obtained from scanned specimens. Specimens were scanned on a Bruker SkyScan 1173 Micro-CT scanner at the Zoological Research Museum Koenig (Bonn, Germany), and on a Phoenix V|tome|x S240 tomograph at Instituto Nacional de Tecnología Industrial (Rafaela, Argentina). Micro-CT data sets were reconstructed using N-Recon software (Bruker Micro-CT) and VG Studio Max 3.0, respectively. The scan parameters for the scanned specimens are detailed in the Supplementary Table S3. Renderization and segmentation of the datasets to separate the skull from vertebrae and the lower jaw was generated using Amira 2.0 software (Thermo Fisher Scientific).

### 3D landmarks and semilandmarks collection, and geometric morphometric analyses

We constructed 3D surface models (.ply files) for skulls and right lower jaw for fifteen individuals of *Philodryas* species (eleven individuals of *P. patagoniensis*, two *P. agassizii*, one *P. psammophideus*, and one *P. varia*) and quantified their shape using geometric morphometrics (Supplementary Table S2). The meshes are freely available on https://www.morphosource.org/projects/000600534?locale=en. Cranial landmarks were chosen to describe biologically meaningful features but also ontogenetically stable^3,14^. In total, 57 landmarks, 30 semilandmarks on 2 curves on both sides of the cranium, and 75 surface semilandmarks were digitized using 3D Slicer (https://www.slicer.org/;^52^). For the lower jaw, only the right counterpart was landmarked; wherein 10 landmarks and 4 curves of semilandmarks were placed. A list and description of all these landmarks are provided in the supplementary information (Table S4), along with graphic representations in Fig. S1. By using the Morpho R package v2.11^53^, an atlas was created to conduct a semiautomated procedure to place the patch of surface semilandmarks (Supplementary Fig. S1) and then transfer it on all skull models.

Curve and surface semilandmarks were allowed to slide along their tangent by minimizing the bending energy^54^ using the Morpho R package 2.11^53^. This package was also used to perform Generalized Procrustes Analysis of the landmark configurations, and to obtain the centroid size (CS, i.e. the square root of the summed squared distances from all coordinates to the centroid configuration*;*^55^ and references cited therein)*.* Furthermore, to provide a measure of overall shape change, the values of Procrustes distance for skull and lower jaw of each specimen were also calculated (Supplementary Tables S5, S6).

A principal component analysis (PCA) was carried out for skulls and lower jaws separately to quantify and visualize the shape variation across the dataset^55^. The function *getMeaningfulPCs* from Morpho package 2.11^53^ was applied to determine whether a PC is entitled to interpretation.

### Ontogenetic dietary survey

To explore the relationship between the morphological and ecological traits of snake feeding, we surveyed information about body size (TL) and prey type in 145 specimens from published works and a publicly available database SquamataBase accessible through the R package^56^ (Supplementary Table S7).

Also, we dissected preserved specimens of *Philodryas patagoniensis* housed in several collections in Argentina (Supplementary Table S7). We identified prey items to the genus level when possible and recorded the total number of items. In case of two or more different items found in gut content, we duplicated the record. A one-way ANOVA and Tukey post-hoc tests were used to identify significant pairwise differences between means of the total length and prey type. Boxplots were plotted using the ggplot2 R package^57^. All described analyses and graphic visualizations were conducted using R version 4.2.3^58^. The R codes used for analyses are deposited in Figshare repository within the project labelled “*Philodryas-*Chuliver & Scanferla 2024” (https://figshare.com/s/a922dcea4ec1a5865ab8).

### Supplementary Information

Supplementary Information 1.

Supplementary Tables.

## Data Availability

All data generated or analysed in this study are included in the main text and Supplementary Information files, and on Morphosource.org under the project name ‘Snake ontogeny and evolution’ (https://www.morphosource.org/projects/000600534?locale=en). The R codes used for analyses are deposited in Figshare repository within the project labelled “*Philodryas-*Chuliver & Scanferla 2024 “(https://figshare.com/s/a922dcea4ec1a5865ab8).
