# Supplementary material for: Paedomorphosis and retention of juvenile diet lead speciation in a group of Neotropical snakes (Colubroides-Philodryadini)
Source: Sci Rep. 2024 May 2;14:10071. doi: 10.1038/s41598-024-60885-y (PMC11066030; doi:10.1038/s41598-024-60885-y)
Supplement: Supplementary file 1 — Supplementary Information 1. [file 41598_2024_60885_MOESM1_ESM.docx]

**Supplementary Information**

**Supplementary Table S1**

Specimens of Philodryadini for which the skull morphology was analyzed. Abbreviations: CFA-RE, herpetological collection of Fundación Azara (Argentina); FML, Fundación Miguel Lillo (Argentina); MACN, Museo Argentino de Ciencias Naturales (Argentina); SMF, Senckenberg Museum Frankfurt (Germany); UMMZ, University of Michigan Museum of Zoology (United States); ZFMK, Herpetological Collection of the Alexander Koenig Museum (Germany).

**Supplementary Table S2**

Specimens for which the skull morphology was analysed through a geometric morphometrics approach. Abbreviations: CFA-RE, herpetological collection of Fundación Azara (Argentina); UMMZ, University of Michigan Museum of Zoology (United States); ZFMK, Herpetological Collection of the Alexander Koenig Museum (Germany).

**Supplementary Table S3**

Scan parameters of the scanned specimens of *Philodryas*. *Philodryas agassizii* CFA-R 811 and *Philodryas patagoniensis* CFA-RE 812 were scanned on a Phoenix V|tome|x S240 tomograph at Instituto Nacional de Tecnología Industrial (INTI), other specimens were scanned on a Bruker SkyScan 1173 Micro-CT scanner at the Zoological Research Museum Koenig (ZFMK).

**Supplementary Table S4**

List and description of the landmarks and curve semilandmarks placed on the skull and lower jaw of the specimens used for 3D geometric morphometric analysis.

**Supplementary Table S5**

Centroid size and Procrustes distances for the skull of each specimen. In red are the highest and lowest values, and highlighted in yellow those values mentioned in the text.

**Supplementary Table S6**

Centroid size and Procrustes distances for the lower jaw of each specimen. In red are the highest and lowest values, and highlighted in yellow those values mentioned in the text

**Supplementary Table S7**

Dietary survey of *Philodryas patagoniensis*, indicating total length, gut content and prey type.

**Supplementary Table S8**

Results of Tukey test for multiple comparisons of means, 95% confidence level. The p values shown correspond to differences between pairs of prey types, being all the values between Arthropods and the remaining types significant.

**Supplementary Figures**


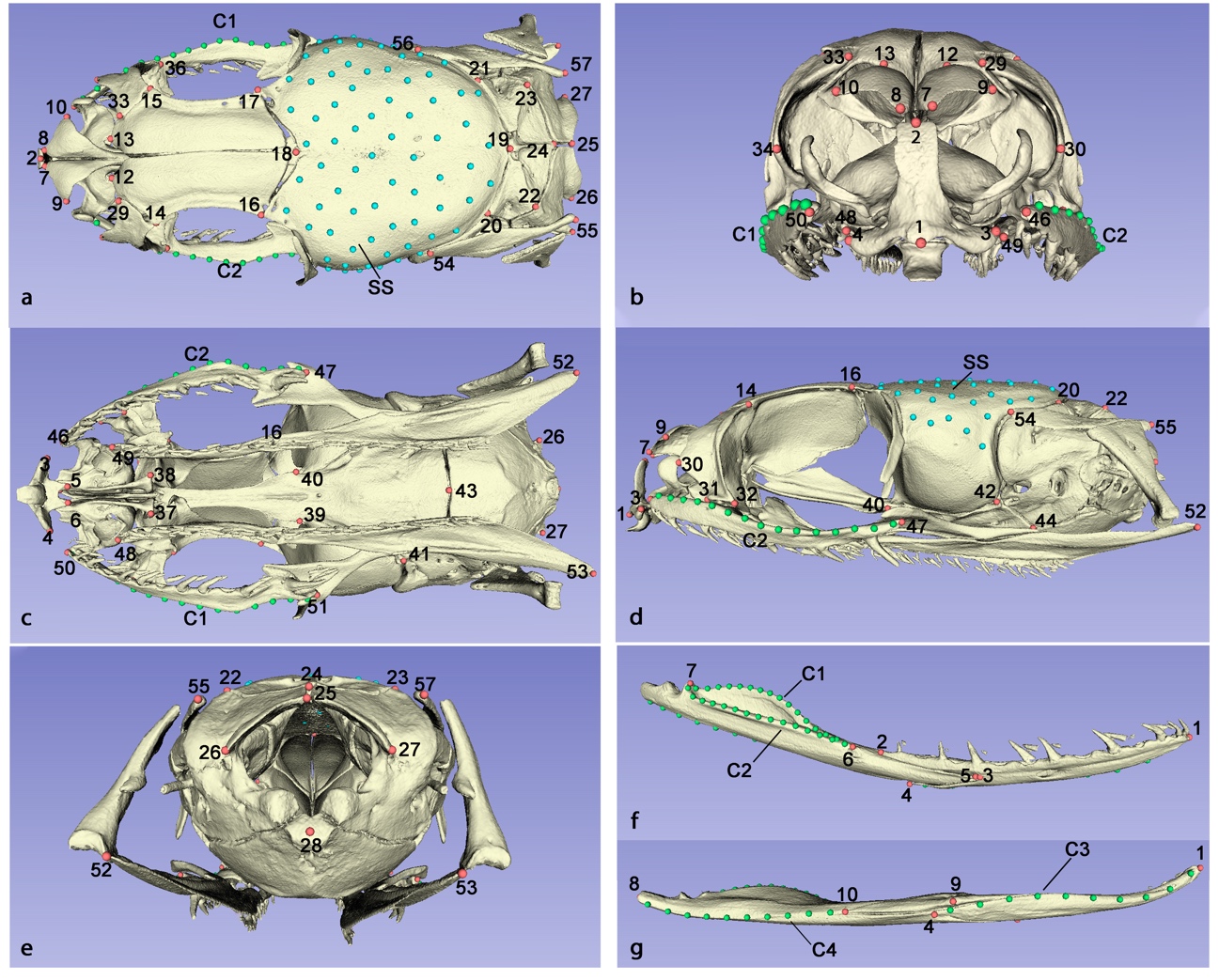


**Figure S1**. Locations for all landmarks and semilandmarks scored in this study, exemplified here by a *Philodryas patagoniensis* individual, showing (a) dorsal, (b) frontal, (c)ventral, (d) lateral, and (e) posterior views of the skull. (f) Laterodorsal and (g) ventral views of the lower jaw. C, curve; SS, surface semilandmarks.


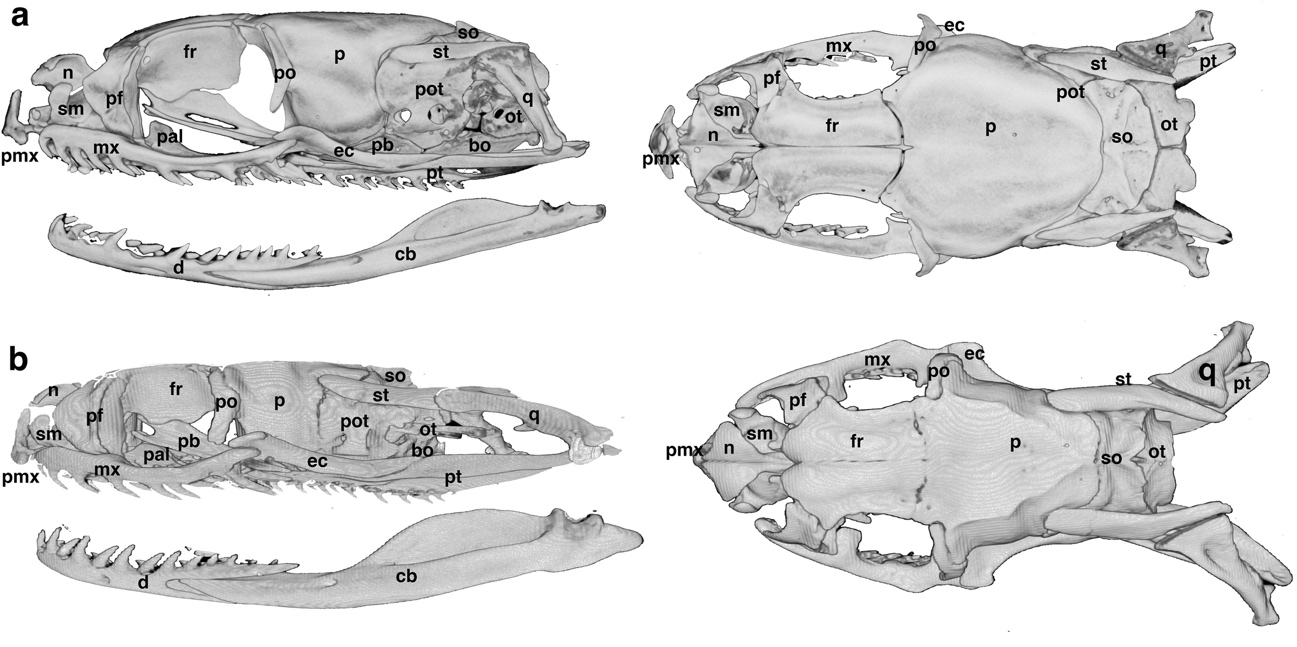


**Figure S2.** Comparison of the skull of species of the *P. patagoniensis* group. (a) juvenile of *Philodryas varia* (TL = 370 mm) and (b) adult of *Philodryas psammophidea* (TL = 830 mm) in lateral (left) and dorsal (right) view. *bo*, basioccipital; *cb*, compound bone; *d*, dentary; *ec*, ectopterygoid; *fr*, frontal; *mx*, maxilla; *n*, nasal; *ot*, otoccipital; *p*, parietal; *pal*, palatine; *pb*, parabasisphenoid; *pf*, prefrontal; *pmx*, premaxilla; *po*, postorbital; *pot*, prootic; *pt*, pterygoid; *q*, quadrate; *sm*, septomaxilla; *so*, supraoccipital; *st*, supratemporal.
